# Application of a Small Unmanned Aerial System to Measure Ammonia Emissions from a Pilot Amine-CO_2_ Capture System

**DOI:** 10.3390/s20236974

**Published:** 2020-12-06

**Authors:** Travis J. Schuyler, Bradley Irvin, Keemia Abad, Jesse G. Thompson, Kunlei Liu, Marcelo I. Guzman

**Affiliations:** 1 Department of Chemistry, University of Kentucky, Lexington, KY 40506, USA; travis.schuyler@uky.edu (T.J.S.); keemia.abad@uky.edu (K.A.); jesse.thompson@uky.edu (J.G.T.); 2 Center for Applied Energy Research, University of Kentucky, Lexington, KY 40511, USA; bradley.irvin@uky.edu (B.I.); kunlei.liu@uky.edu (K.L.); 3 Department of Mechanical Engineering, University of Kentucky, Lexington, KY 40506, USA

**Keywords:** ammonia emission, monoethanolamine, amine scrubbing, coal, flue gas, unmanned, UAV, UAS, post-combustion CO_2_ capture

## Abstract

The quantification of atmospheric gases with small unmanned aerial systems (sUAS) is expanding the ability to safely perform environmental monitoring tasks and quickly evaluate the impact of technologies. In this work, a calibrated sUAS is used to quantify the emissions of ammonia (NH_3_) gas from the exit stack a 0.1 MWth pilot-scale carbon capture system (CCS) employing a 5 M monoethanolamine (MEA) solvent to scrub CO_2_ from coal combustion flue gas. A comparison of the results using the sUAS against the ion chromatography technique with the EPA CTM-027 method for the standard emission sampling of NH_3_ shows good agreement. Therefore, the work demonstrates the usefulness of sUAS as an alternative method of emission measurement, supporting its application in lieu of traditional sampling techniques to collect real time emission data.

## 1. Introduction

The use of small unmanned aerial systems (sUAS) has expanded significantly in the scientific community over the last few years. Recent studies have demonstrated the usefulness of sUAS for providing novel measurements in the boundary layer that were previously infeasible with existing methods (i.e., ground stations, towers, weather balloons, manned aircraft, satellites) [1,2,3]. These studies illustrate several advantages that motivate the incorporation of sUAS for atmospheric research. The ability to provide nearly instant real-time emission estimates without the traditional lead times that accompany wet-lab analysis, such as ion chromatography (IC), is desirable in environmental and industrial applications. Furthermore, from the operational cost perspective, the total investment needed for the sUAS is far cheaper than that for costly instrumentation with its associated consumables and periodic services [1]. Additionally, from the safety viewpoint, there is considerable risk associated with sampling gases from hard-to-access stacks for off-line analysis, which also require continuous human checks. Thus, through a systematic flight plan sUAS can easily mitigate the previous sampling hazards. The occasional replacement of batteries is the primary upkeep cost of the sUAS, therefore it is a cost-effective technology to purchase and maintain. Because of the widespread pollution of anthropogenic carbon dioxide (CO_2_), coal-burning powerplants are a relevant case study for the industrial applications of sUAS for the quantification of point source pollution.

Coal burning power plants emit 4.90 × 10^3^ Tg of carbon dioxide (CO_2_) a year, accounting for ~21% of all CO_2_ emitted throughout the United States of America (U.S.A.) in the year 2017 [4]. It is well-known that CO_2_ gas absorbs infrared radiation and traps heat in the Earth’s atmosphere, working as a greenhouse gas [5]. As a result, continued anthropogenic CO_2_ pollution increases radiative forcing, leading to an increase in the average global surface temperature [5]. Thus, practical technologies that counteract the projected increase in CO_2_ emitted are needed, which should not significantly change the existing methods of energy production [6]. There are numerous strategies to mitigate the effect of fossil fuel-burning power plants and counteract pollution due to the constantly amplifying demand for energy in the last few decades [6,7]. An effective way to reduce emissions in existing coal burning power plants is to retroactively fit (retrofit) them with carbon capture systems (CCS) [8,9] which trap and remove CO_2_ from the flue gas [10]. One successful solution employs a post-combustion treatment of the flue gas via the absorption of the CO_2_ with a circulating amine solvent, with a potential emission reduction of up to 90% [11,12,13,14,15,16,17]. Many of the 359 coal burning power plants that operate across the U.S.A. [18] can potentially be fitted with CCS technology. Currently, 65 large-scale projects have started retrofitting carbon capture technologies in the U.S.A. [19].

Ammonia (NH_3_) gas can be produced from a variety of sources, and has been identified in low quantities as a byproduct from amine-based CCS as a result of the oxidative degradation of the absorbing solvent [20,21,22,23,24]. NH_3_ is a pollutant with undesirable chemical effects in the atmosphere [25], where large levels can promote aerosol chemistry [26], which affects air quality and climate by forming particulates that are detrimental to public health and scatter solar radiation, respectively [27,28]. For example, the presence of NH_3_ above background levels can change nitrate aerosol chemistry [29]. Therefore, accurately measuring the emissions of NH_3_(*g*) from reported sources is of significant importance. Herein, we quantify NH_3_(*g*) emissions from a small pilot-scale CCS with a coal-fired flue gas generator using a 5 M (30.0 wt.%) monoethanolamine (MEA) solvent. For this study, we used the UKySonde, a sUAS [1] equipped with a calibrated NH_3_(*g*) sensor [2], along with standard gas emission sampling methodology for comparison.

## 2. Materials and Methods

### 2.1. CO_2_ Capture System

**Figure 1:**
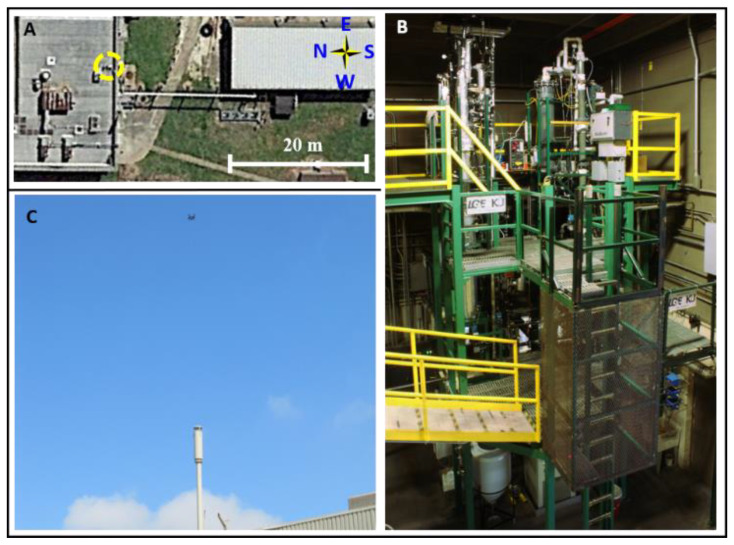
(**A**) Aerial view of the facilities employed in the University of Kentucky Center for Applied Energy (UKy-CAER). The coal flue gas generator is located to the right side and the gas flows through a polyvinyl chloride (PVC) pipe (from right to left) to the building on the left-hand side for amine treatment in the carbon capture system (CCS) before the scrubbed flue gas emissions are released out the stack at the top (yellow dashed circle). (**B**) Image of the amine-based CCS at UKy-CAER. (**C**) Perspective view of the small unmanned aerial system (sUAS) flying above the stack.

The University of Kentucky Center for Applied Energy (UKy-CAER) built and operates a bench scale 0.1-megawatt thermal (MWth) CO_2_ capture system using a coal burning flue gas generator (FGG) to provide representative coal-derived flue gas to the CCS (10–14% CO_2_, 8–10% O_2_, other trace gases, and balanced with N_2_). The UKy-CAER CSS is typically operated during normal working hours in a manner to replicate extreme operation conditions (e.g., high temperatures and contaminate levels) to accelerate solvent degradation and obtain meaningful data form a short operational number of hours. The produced flue gas is transported to the building housing the CCS in the left-hand side of Figure 1a, where 5 M of MEA solvent (in water) is used in a typical aqueous amine-based CCS absorber/regeneration configuration (Figure 1b) to separate the CO_2_ from the flue gas. Finally, the treated flue gas is released out of a stack (0.194 m diameter), which is in the center of the yellow circle in Figure 1a. All the NH_3_ concentrations reported from the sUAS readings are measured in close proximity to the stack (as explained below) to model the source point. The scrubbed flue gas typically contains > 2% CO_2_, along with NH_3_(*g*) from the degradation of the amine solvent. The NH_3_(*g*) concentration is solvent- and regeneration temperature-dependent. The total emissions from this CCS are relatively small and well below thresholds considered significant from an air permitting perspective [30]. The NH_3_(*g*) emissions in the scrubbed flue gas were quantified using an sUAS (Figure 1c), with comparison to the daily emission average values collected using a standard stack sampling methodology based on ion chromatography with the method EPA CTM-027 [20,30]. The experiments with the sUAS took place between September 10 and 14, 2018, above the stack exhaust. The measurements of the background NH_3_(*g*) mixing ratio, defined in this work as the mole fraction in air [27], were completed at the ground level (away from the coal burning FGG and CCS) on September 10. The NH_3_(*g*) mixing ratios above the stack were registered from September 11 to 14, 2018. The bench facility was fully operational on September 10, 11, and 13, 2018. The standard EPA CTM-027 method was used to compare the sUAS measurements and demonstrate the ability of the sUAS to replace conventional measurement techniques.

### 2.2. Quantification of NH_3_(g) with the UKySonde

In the sUAS, called the UKySonde, an analytical sensor (MICS-6814 sensor) is integrated to measure the mixing ratio of NH_3_ with a 3DR Solo quadcopter from 1 to 2 m above from the stack located at 20 m above ground level (AGL). A 10-bit Arduino UNO microcontroller (Somerville, MA, USA) with a V2 Base Shield (SEEED Studio, Nanshan, China) and an Arduino Wireless SD Shield operating at 5.0 V were used to control the sensing package [2]. Thunder Power batteries with 1350 mA h capacity (RC 2S, Las Vegas, NV, USA) provided the power for data collection, which was set at a rate of 1 Hz, recorded in a SanDisk Ultra Class 10 microSD card with 8 GB capacity (Milpitas, CA, USA). The sensor was calibrated in a custom environmental chamber using standards from 5.00 ppbv to 90.00 ppmv, as described previously [2]. The corresponding linear regression through the origin for this calibration curve is described by Equation (1):

Mixing Ratio of NH_3_ (ppmv) = 1.0085 × Sensor Response (ppmv). (1)

The performed calibration expanded the operating range of the sensor (from factory specification) to sub-ppmv levels, with an accuracy within ±0.20% of the measured value and a precision of 30 ppbv. Programed flights with Mission Planner Autopilot software were used to monitor the NH_3_(*g*) levels at a rate of 1 Hz during operation every 1 h starting at 9 AM (UTC-5). The flights were designed to measure NH_3_(*g*) directly from 1 to 2 m above the exhaust stack, corresponding to the maximum mixing ratio in the flue gas after the CCS. The mixing ratios of NH_3_ reported below for measurements from 1 to 2 m above the stack were corrected after subtracting the average background measured when the fired furnace (located ~60 m downwind) was not operating. Previous work with a tethered balloon equipped with the same sensor package discarded any prop-wash effect by the quadcopter simultaneously measuring NH_3_ for altitudes between 10 and 120 m above the ground level [2]. The sensor was allowed to warm-up and equilibrate for at least 1 h before take-off operation of programed flights with Mission Planner Autopilot. The flight plan was programed to reach an altitude of 140 m AGL, travel horizontally to the top of the stack position, descend to an altitude of 21 m AGL, and registering data for reporting during the ascent from 21 to 22 m AGL (from 1 to 2 m above the top of the exhaust stack). For data recovery, the device was powered down before removing the SD card.

### 2.3. Conventional Ammonia Emission Sampling

Ammonia emissions were collected by adapting the methodology from U.S.A. EPA CTM-027 to the facilities 7.62 cm diameter flue gas exit pipe, designed for this purpose, within the CCS. In this method, gaseous ammonia is withdrawn from the CCS exit stack and collected in an aqueous dilute sulfuric acid solution inside a four impinger train connected to a pump (flow rate of 4 L min^−1^) and metering console. Sulfuric acid was purchased (VWR, Wayne, PA, USA) pre-diluted to the target concentration of 0.05 M. The impinger solutions were combined and stored at 4 °C prior analysis. The method typically samples > 500 L of gas for each sample, with an average of around 600 L of gas and a collection time of 2 h. Field blanks were collected in parallel during the sample collection period as a measure of quality control and ambient ammonia levels and were always below the analytical detection limit. Ammonia absorbed into the sulfuric acid impinger solutions was analyzed by ion chromatography (IC). The samples were diluted with 18.2 MΩ cm^−1^ water and analyzed by cation ion chromatography using a Dionex ICS-3000 system with an AS autosampler (Dionex-Thermo Scientific, Sunnyvale, CA, USA). The cation IC system consisted of a CSRS 300 suppressor, EGC III MSA (methanesulfonic acid, MSA) eluent generator, IonPac CS17 analytical column and a CG17 guard column operated at 30 °C. The conductivity detector was maintained at 35 °C with a sample injection volume of 20 μL. An isocratic mobile phase of MSA (2.5 mM) at a flow rate of 0.8 mL min^−1^ was used. Ammonia quantitation used a calibration curve made from a stock ammonium (NH_4_^+^) solution (Environmental Express, Charleston, SC, USA). Peak identification and integration was performed manually using the Dionex Chromeleon chromatography management system software version 6.80. Each sample was analyzed in triplicate and the average value was used to calculate the gas phase analyte concentration in ppmv [30]. The method typically requires ~2 h for sample collection and ~1 h for actual analysis per sample.

### 2.4. Emission Estimates of NH_3_(g)

The mixing ratio of NH_3_ (in ppmv), measured immediately above the exhaust stack, were integrated over time and combined with the flow rate of the treated flue gas at the exhaust of ~14.7 actual cubic feet per minute (acfm) to obtain the mass flow of emissions with the industry standard of pounds of NH_3_ per day (lbs day^−1^). The average mass flow measured for each flight 1 m above the exhaust stack was fed into a Gaussian plume model to retrieve the mixing ratio inside the stack prior to diffusion. For this model, the atmospheric conditions were neutral, and the exit velocity and temperature of the flue gas were 0.232 m s^−1^ and 40 °C, respectively. The specific wind inputs for each flight were provided by the nearest weather station (KKYGEORG28) at Horse Country, ~3.2 km away [31].

## 3. Results and Discussion

The UKySonde flew every ~1 h during 8 h from 11 to 14 September 2018, measuring the NH_3_(*g*) levels from the flue gas after treatment with MEA in the CCS on September 11 and 13, and the background levels (with the furnace off) on September 12 and 14. The NH_3_(*g*) mixing ratio attributed to the UKy-CAER CCS can be retrieved from the average mixing ratio of NH_3_ measured in close proximity to the stack throughout the day. The measurement of NH_3_(*g*) in the flue gas using the standard stack sampling method took place under generally the same CCS operating conditions, but not in parallel with sUAS sampling. Daily averages of NH_3_(*g*) emissions were quantified and used to compare with the sUAS measurements.

### 3.1. Ammonia Measurements

**Figure 2:**
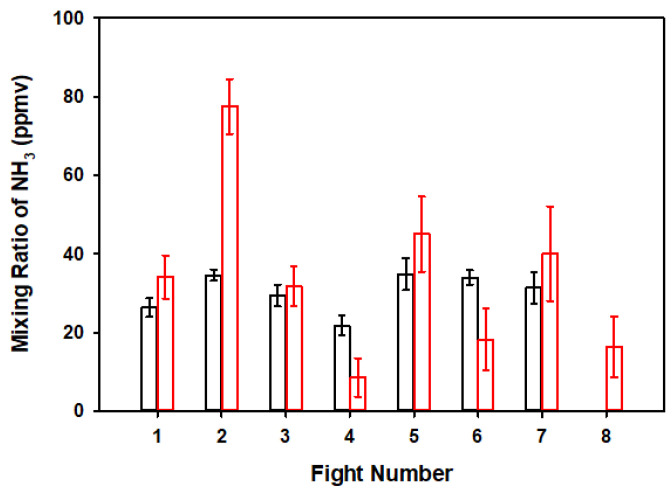
Mixing ratio of NH_3_(*g*) measured 1 m above the exhaust stack every 1 h (from 9:00 AM, UTC-5) by the UKySonde on September 11 (**black**) and 13 (**red**) of 2018.

The background mixing ratio determined between 12 and 4 pm on September 10th, was 7.26 (±0.35) ppbv. Figure 2 shows the measurements of NH_3_(*g*) on 11 and 13 September, which took place every 1 h. The average mixing ratios of NH_3_ 1 m above the stack (at the virtual point source) was 28.90 (±7.60) ppmv on September 11 (black columns in Figure 2). Similarly, during September 13 (red columns in Figure 2), the average 26.70 (±8.80) ppmv NH_3_(*g*). On 12 and 14 September, the CCS was not operational, and the flights completed with the UKySonde measured a background mixing ratio 1 m above the exhaust stack, near the low calibration limit of 6.93 (±2.28) and 6.95 (±1.57) ppbv NH_3_(*g*), respectively.

### 3.2. Gaussian Modeling of the Point Source

The average mixing ratio measured in each flight (Figure 2) was fed into a Gaussian plume model to retrieve the mixing ratio inside the stack prior to diffusion. The Gaussian plume model was chosen to estimate the concentration inside the stack based on measurements taken by the sUAS ~1 m above the stack exit [32,33,34,35]. The model was chosen due to its proven performance over small distances. In other words, this model has been used on small scales on many occasions and successfully modeled the dispersion of gas molecules [36,37,38]. The model was effectively applied to NH_3_(*g*), as the measurements of this pollutant were completed under stable meteorological conditions for a short distance in less than 1 h, which is much shorter than its atmospheric lifetime, $\tau_{{\mathrm{NH}}_{3}\left(g\right)}$ = 1 day [28,39].

The diffusion of flue gas from the exhaust stack to the NH_3_(*g*) sensor depends on many factors, including the emission rate, distance from source, and atmospheric conditions that influence plume characteristics. The most significant atmospheric conditions considered are wind speed, wind direction, and boundary layer stability, all parametrized in the Gaussian plume model to analytically retrieve the original point source concentration. The estimated NH_3_(*g*) emission uncertainly is propagated through the sUAS measurements and carried into the model. The overall uncertainty of the measurements is propagated to the final output of the model.

The Gaussian plume model makes the following five assumptions: (1) continuous emission with negligible background pollution; (2) chemical stability, mass conservation after surface contact and negligible deposition; (3) steady-state meteorological conditions, negligible change in wind speed and direction with time and altitude; (4) dispersion parameters are a function of horizontal distance, negligible diffusion in the direction of travel; and (5) the topography does not alter the plume.

**Figure 3:**
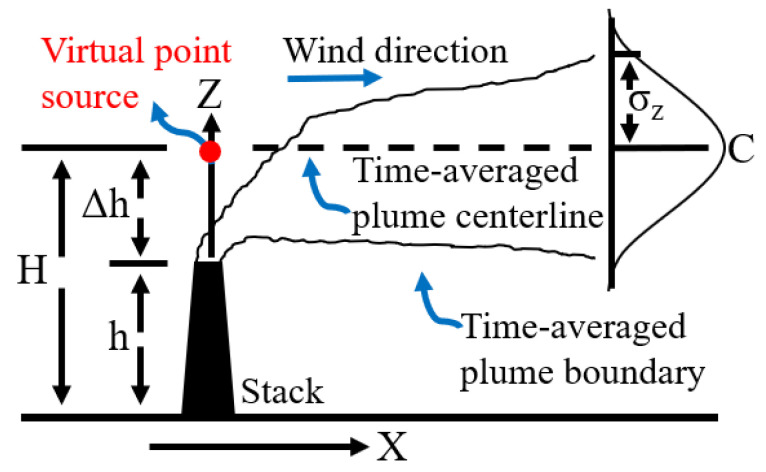
Schematic representation of the Gaussian plume model utilized to retrieve the mixing ratio of NH_3_(*g*) in the exhaust stack from the values measured 1 m above.

The Gaussian dispersion Equation (2) provides the concentration, *q_e_,* which depends mainly on the initial flux determined by the UKySonde, *F_e_*, the wind speed oriented along the *x*-axis, *u*, and CCS flow rates, and the dispersion coefficients, σ_y_ and σ_z_ in the respective directions *y* (horizontal and perpendicular to *x*) and *z* (vertical to *x*)*,* which define the spread of the plume. As for any normal distribution, a 67% of the emitted plume of NH_3_(*g*) falls within ±σ*_y_* and ±σ*_z_* in each direction. The stable atmospheric conditions during all measurements are properly fit with small dispersion coefficient values. Thus, as the *x*-axis is defined in the direction of the wind associated with the diffusion coefficient σ_x_, the horizontal σ_y_ is largely dependent on crosswinds of variable speed and direction in the *y*-axis. The vertical distance from the source, relative to the point source height, represented in the *z* direction in Figure 3, is associated with a vertical diffusion coefficient, σ_z_, which varies with temperature gradients. Stable atmospheric conditions reduce diffusion, resulting in small σ_z_ values.

(2) $$q_{e}=\frac{F_{e}}{{2\pi \sigma }_{y}\sigma_{z}u}\exp\left(\frac{-(y{)}^{2}}{{2\sigma }_{y}^{2}}\right)\left\{\exp\left(\frac{-(z\;-\;H{)}^{2}}{2\sigma_{z}^{2}}\right)+\exp\left(\frac{-(z\;+\;H{)}^{2}}{{2\sigma }_{z}^{2}}\right)\right\}.$$

Table 1 shows the input parameters used in the model for each flight, where the indicated input NH_3_ (ppmv) was used to retrieve the flux of the emitted NH_3_ (µg/s) needed at the point source to create the measured amounts at the virtual point (1 m above). The flux emitted was finally converted into a model mixing ratio in ppmv units. For the previous purpose, the exit gas velocity transported through the fixed stack surface area at the exhaust was needed. As an example of the data treatment for the flights, the model ran with atmospheric conditions that were neutral, and the exit velocity and temperature of the flue gas were 0.232 m s^−1^ and 40 °C, respectively. The stack diameter was 0.194 m. The specific wind inputs and ambient air temperatures for each flight were provided by the nearest weather station (KKYGEORG28) at Horse Country, ~3.2 km away. The wind speed for each flight was constant (e.g., for the first flight on September 11 it was consistently at 7 mph NE, with a temperature of 20.0 °C (Table 1)). The Gaussian plume model run as a function of distance from the stack (~ 1 m) was used to estimate the typical σ_y_ and σ_z_ values. The wind speed on September 11 was consistent for all flights, except for flight 4, which slowed down by less than 5%. Instead, the windspeeds on September 13, although lower than for September 11, varied by 52% among the eight flights. This variance in wind speed affecting plume dispersion contributed the most significant source of uncertainty introduced by the model. Table 1 also sows the results of converting the output mixing ratio in ppmv units into an emission flux with units of lbs/day.

The quantification of the NH_3_(*g*) in air above the exhaust stack is not affected by any chemical transformations during the short distance from the exit orifice and the time scale of measurements. However, in addition to physical variability—i.e., provided by wind speed and temperature—the sensor registers any fluctuations in the NH_3_(*g*) emitted due to changes in the chemical composition of the treated flue gas due to dynamic MEA decomposition reactions. Thus, although the exit gas velocity from the exhaust stack in Table 1 remains constant, the composition of gas varies as recorded by the UKySonde measurements combined with the Gaussian plume model.

### 3.3. Comparison of NH_3_(g) Emissions Measurement Techniques

Table 1 also displays the model output data for each flight in Figure 1, which indicate that the average mixing ratios of NH_3_ measured at the virtual point during September 11 and 13, 28.90 (±7.60) ppmv and 26.70 (±8.80) ppmv, correspond to the mixing ratios at the point source (inside the stack) of 80.30 (±10.10) ppmv and 82.00 (±13.30) ppmv, respectively. The retrieved mixing ratios from the modeled UKySonde measurements are in close agreement with the ion chromatography results of seven samples collected in the exhaust from the CCS, which were in the range from 11.90 to 120.0 ppmv, with an average of 84.40 (±18.00) ppmv. In addition, the retrieved point source mixing ratios can be easily converted to emission rates of 7.20 (±0.93) × 10^−3^ and 7.55 (±1.23) × 10^−3^ lbs day^−1^ of NH_3_(*g*) on September 11 and 13, respectively.

The mixing ratio of NH_3_ was measured with two independent analytical methods, providing quality assurance of the similar NH_3_(*g*) emission reported by the UKySonde and the standard stack sampling method during the operation of the CCS. The combined sUAS method with the Gaussian plume model determined an average NH_3_ mixing ratio of 81.20 (±16.70) ppmv in the flue gas, while conventional gas sampling measured an average of 84.40 (±18.00) ppmv. Both values agreed reasonably well within the experimental errors expected from two different analytical techniques. These measured values are also comparable to published ammonia emission values from the MEA testing campaigns at other CCS units of variable size and flue gas composition [30]. The overlapping results of the emission estimates from the traditional sampling methods and the sUAS further supports their use in quantifying industrial point source pollution. This work demonstrates that the integration of sUAS into routine emission sampling can provide accurate, real-time results and can be a valid alternative to wet-lab techniques.

## 4. Conclusions

Several advantages and disadvantages can be considered for applying the sUAS method in this work for determining NH_3_(*g*) emissions as compared to the traditional offline EPA ion chromatography sampling method. The new method is cheap and easy to implement while providing a faster measurement and sampling frequency than ion chromatography. In addition, the use of a sUAS eliminates the risk to human life associated with sample collection requiring the climbing of hard-to-access exhaust stacks emitting toxic pollutants. However, the use of a sUAS requires access to meteorological information or the ability to register it. In this context and as a disadvantage, other atmospheric regimes than the one studied here would require the implementation of alternative dispersion models and in situ wind measurements. The implementation of the technique also requires a licensed sUAS pilot and appropriate flying authorizations. A final potential advantage of the sUAS is to allow for the simultaneous sampling of other pollutants with appropriate sensor probes and emission parameters by including sonic anemometers for determining wind speed and direction and physical parameters (temperature, pressure, and relative humidity).

Currently, the total annual NH_3_(*g*) emissions in the U.S.A. is 3.0 (± 0.2) Tg year^−1^, with 70–80% attributed to agriculture (e.g., fertilizer and manure) and the remaining 20–30% originating from industry and other anthropogenic sources [40]. Having the ability to quickly measure NH_3_(*g*) emissions from a point source, such as this amine-based CCS, has broad implications and is a potential application of this technique. The ability to record essentially real-time emission data is of significant interest to utility and industrial locations, in addition to regulatory agencies. Overall, this work demonstrates that NH_3_(*g*) emissions can be estimated with confidence using sUAS and are a viable option to replace existing sampling methods. The use of sUAS as an analytical tool to quantify point source anthropogenic pollution NH_3_(*g*) can find application also in natural settings. In this regard, future work could be expanded by performing horizontal flight patterns with in situ wind measurements to characterize the plume completely as it traverses the boundary layer and undergoes atmospheric ageing. Possible interesting efforts in this field could also include numerical simulations to compare with present or new measured results. Particularly, the recent development of computational fluid dynamics (CFD) numerical simulations can contribute to improve the analysis of pollutant emissions and their spread [41,42]. Finally, this work also serves as an example for future applications of sUAS to monitor pollution emissions—i.e., in the case of agriculture and ammonia contributed by livestock or from rice paddies.

## Figures and Tables

**Table 1:** Input parameters for the Gaussian plume modeling and results.

Date	Flight #	Mixing Ratio of NH_3_(*g*) (ppmv)	Stack Radius (m)	Exit Gas Velocity (m/s)	Exit Gas Temp (°C)	Air Temp (°C)	Wind Speed (m/s)	Emitted NH_3_ (µg/s)	Model Output NH_3_(*g*) (ppmv)	NH_3_(*g*) Emission (lbs day^−1^)
9/11/2018	1	26.28 (±2.40)	0.097	0.232	40	20.0	3.1	667	68	6.27 (±0.59) × 10^−3^
	2	34.54 (±1.43)	0.097	0.232	40	20.0	3.1	877	90	8.29 (±0.35) × 10^−3^
	3	29.38 (±2.81)	0.097	0.232	40	20.6	3.1	746	77	7.10 (±0.69) × 10^−3^
	4	21.73 (±2.58)	0.097	0.232	40	21.1	2.7	552	65	5.99 (±0.63) × 10^−3^
	5	34.82 (±4.07)	0.097	0.232	40	21.7	3.1	884	91	8.39 (±1.00) × 10^−3^
	6	33.96 (±1.81)	0.097	0.232	40	21.7	3.1	863	89	8.20 (±0.44) × 10^−3^
	7	31.34 (±3.97)	0.097	0.232	40	20.6	3.1	796	82	7.56 (±0.97) × 10^−3^
9/13/2018	1	34.09 (±5.50)	0.097	0.232	40	28.9	1.0	247	105	9.66 (±0.31) × 10^−3^
	2	77.50 (±6.94)	0.097	0.232	40	30.0	1.0	878	238	2.19 (±0.39) × 10^−3^
	3	31.67 (±5.00)	0.097	0.232	40	31.1	1.3	230	75	6.90 (±0.28) × 10^−3^
	4	8.53 (±4.91)	0.097	0.232	40	31.1	3.6	552	10	0.92 (±0.27) × 10^−3^
	5	45.00 (±9.57)	0.097	0.232	40	31.1	1.0	326	138	12.69 (±0.55) × 10^−3^
	6	18.18 (±7.89)	0.097	0.232	40	31.1	2.7	132	21	1.93 (±0.44) × 10^−3^
	7	40.00 (±12.08)	0.097	0.232	40	29.4	2.7	290	46	4.23 (±0.67) × 10^−3^
	8	16.28 (±7.71)	0.097	0.232	40	26.7	2.2	118	23	2.12 (±0.43) × 10^−3^
